# Contrast-enhanced ultrasonography in the ICU: promising tool or exciting toy?

**DOI:** 10.1186/cc12123

**Published:** 2013-03-19

**Authors:** I Göcze, R Herzog, BM Graf, A Agha, HJ Schlitt, K Pfister, E Jung, T Bein

**Affiliations:** 1 University Medical Centre Regensburg, Germany

## Introduction

Contrast-enhanced ultrasonography (CEUS) is a dynamic digital ultrasound-based imaging technique, which allows quantification of the microvascularisation up to the capillary vessels. As a novel method for assessment of tissue perfusion it is ideally designed for use in the ICU. CEUS is cost-effective and safe and can be repeatedly performed at the bedside without radiation and nephrotoxicity.

## Methods

The frequency of CEUS use in the multidisciplinary surgical ICU was retrospectively evaluated for the period from 1 September 2011 to 1 September 2012. Furthermore, contributions of this novel method to the management of critically ill ICU patients as well as its accuracy were assessed.

## Results

In total, 33 CEUS studies were performed in critically ill ICU patients. The most frequent indications included: assessment of the liver perfusion, assessment of the pancreas and kidney perfusion after pancreas and kidney transplantation, assessment of the renal perfusion in acute kidney injury (AKI), assessment of active bleeding and assessment of the bowel perfusion. In all studies, the correct diagnosis was achieved and the transport of critically ill patients to the radiology department for further diagnostic procedures as well as application of iodinated contrast agents was avoided. In 16 cases significant new findings were detected. Twelve of them were missed by conventional standard Doppler ultrasound prior to CEUS. In assessment of seven cases with AKI, impaired or delayed perfusion and microcirculation of the kidney was observed in six patients. In three patients urgent surgical intervention was performed because of CEUS results. In three cases active bleeding was excluded at the bedside due to absence of contrast agent extravasation into hematoma (thigh and perihepatic) or into abdominal cavity, without need for complementary CT imaging or angiography. In one case the regular perfusion of intestinal anastomosis was confirmed with no need for surgical exploration. None of patients undergoing CEUS manifested any adverse reactions or developed any complications associated with the imaging technique.

## Conclusion

Contrast-enhanced ultrasonography clearly improves visualization of the perfusion in various tissues. It is very likely to be superior to standard Doppler ultrasound, and is safe and well tolerated in critically ill patients. Promising indications for the use of CEUS in the ICU may be the assessment of kidney microcirculation and assessment of liver perfusion in liver transplant and liver trauma patients.

